# EM-PLA: environment-aware heterogeneous graph-based multimodal protein–ligand binding affinity prediction

**DOI:** 10.1093/bioinformatics/btaf298

**Published:** 2025-05-12

**Authors:** Zhiqi Xie, Peng Zhang, Zipeng Fan, Qingpeng Zhang, Qianxi Lin

**Affiliations:** College of Intelligence and Computing, Tianjin University, Tianjin 300072, China; College of Intelligence and Computing, Tianjin University, Tianjin 300072, China; College of Intelligence and Computing, Tianjin University, Tianjin 300072, China; Musketeers Foundation Institute of Data Science and the Department of Pharmacology and Pharmacy, LKS Faculty of Medicine, The University of Hong Kong, Hong Kong SAR 999077, China; College of Intelligence and Computing, Tianjin University, Tianjin 300072, China

## Abstract

**Motivation:**

Predicting protein–ligand binding affinity accurately and quickly is a major challenge in drug discovery. Recent advancements suggest that deep learning-based computational methods can effectively quantify binding affinity, making them a promising alternative. Environmental factors significantly influence the interactions between protein pockets and ligands, affecting the binding strength. However, many existing deep learning approaches tend to overlook these environmental effects, focusing instead on extracting features from proteins and ligands based solely on their sequences or structures.

**Results:**

We propose a deep learning method, EM-PLA, which is based on an environment-aware heterogeneous graph neural network and utilizes multimodal data. This method improves protein–ligand binding affinity prediction by incorporating environmental information derived from the biochemical properties of proteins and ligands. Specifically, EM-PLA employs a heterogeneous graph neural network (HGT) with environmental information to improve the calculation of non-covalent interactions, while also considering the interaction calculations between protein sequences and ligand sequences. We evaluate the performance of the proposed EM-PLA through comprehensive benchmark experiments for binding affinity prediction, demonstrating its superior performance and generalization capability compared to state-of-the-art baseline methods. Furthermore, by analyzing the results of the ablation experiments and integrating visual analyses and case studies, we validate the rationale of the proposed method. These results indicate that EM-PLA is an effective method for binding affinity prediction and may provide valuable insights for future applications.

**Availability and implementation:**

The source code is available at https://github.com/littlemou22/EM-PLA.

## 1 Introduction

The binding affinity between proteins and ligands is crucial in drug design, reflecting the interaction strength and serving as a key metric for evaluating drug candidates (Perola 2010). Accurate prediction is essential for drug screening and discovery (Lim *et al.* 2021). In recent years, deep learning has revolutionized this field by effectively extracting complex features from large datasets. This advancement has led to promising structure-based and sequence-based approaches for predicting protein–ligand binding affinity (Zhao *et al.* 2020, Niazi and Mariam 2023, Singh *et al.* 2024, Zhang *et al.* 2024b).

Sequence-based models treat proteins as text sequences, enabling predictions without requiring high-quality structural measurements (Zhao *et al.* 2024). Notable examples include FusionDTA (Yuan *et al.* 2022), DeepDTAF (Wang *et al.* 2021), and CAPLA (Jin *et al.* 2023). DeepDTAF uses protein and pocket sequences along with SMILES representations of ligands for binding affinity predictions, while CAPLA combines protein sequence features with secondary structure information and compound features. However, despite the significant influence of three-dimensional structures on binding strength (Gilson and Zhou 2007), existing sequence-based methods overlook these structural effects, relying on attention mechanisms that may compromise prediction accuracy (Karimi *et al.* 2019, Zhao *et al.* 2022a,b). In contrast, structure-based methods utilize three-dimensional structural information through 3D Convolutional Neural Networks (3D-CNNs) (Ji *et al.* 2013) or Graph Neural Networks (GNNs) (Scarselli *et al.* 2004) to extract features (Zhang *et al.* 2024b). Examples include Pafnucy (Stepniewska-Dziubinska *et al.* 2018), OnionNet (Zheng *et al.* 2019), and ELGN (Yi *et al.* 2024) enhances feature extraction by incorporating long-range interactions or using equivariant GNNs for robust learning. However, these methods often neglect the complete set of amino acids, leading to a loss of protein information (Xu *et al.* 2024), and they struggle with the high computational costs of large-scale molecular graph feature learning (Choi *et al.* 2022). Additionally, they do not differentiate between protein and ligand atoms or between covalent and non-covalent interactions. Covalent interactions occur between atoms within proteins or ligands, while non-covalent interactions take place between protein and ligand atoms (Li *et al.* 2021, Yang *et al.* 2024). This oversight can hinder the accuracy of models in predicting protein–ligand behavior.

**Figure 1.**
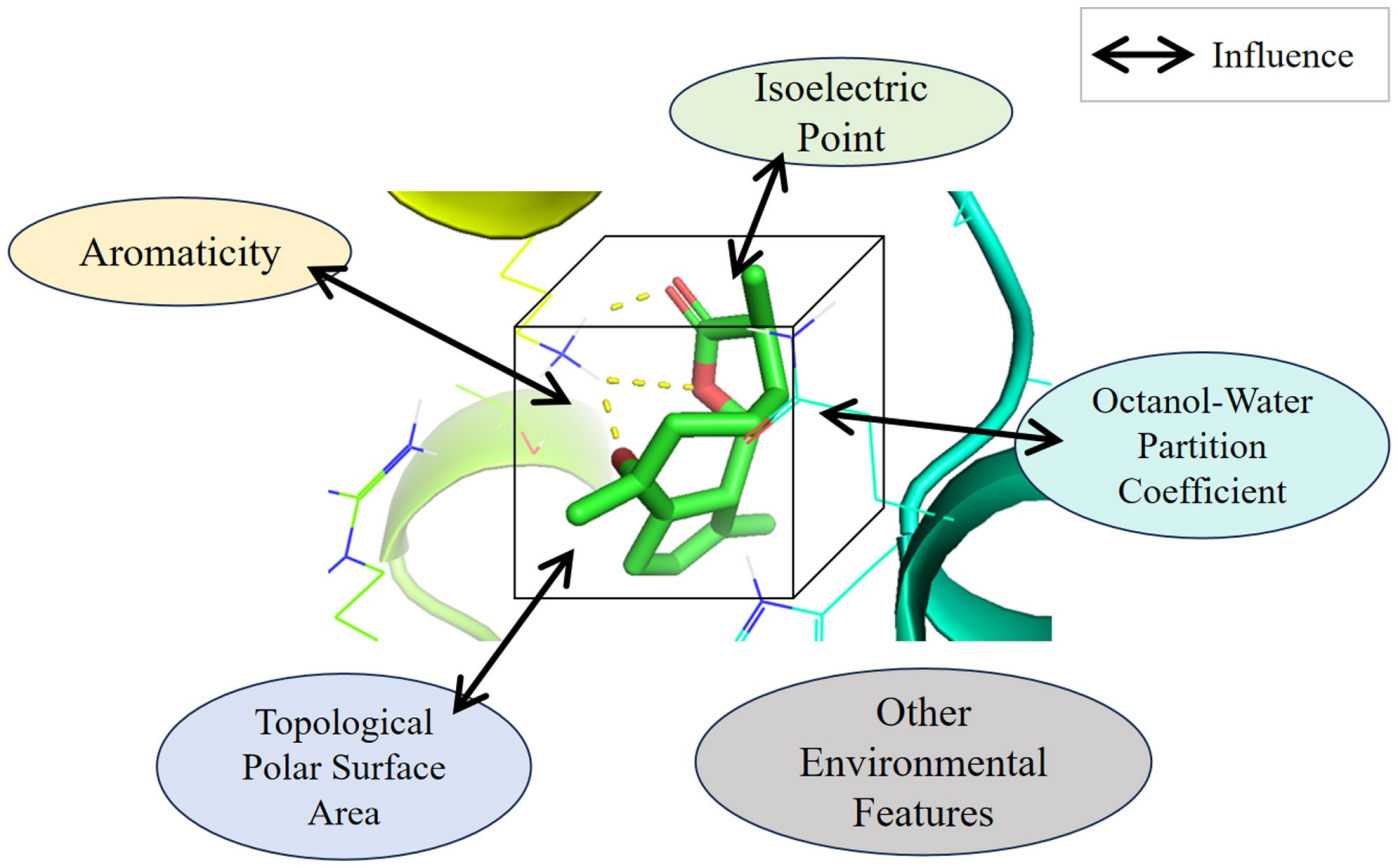
Various environmental features influence protein–ligand binding affinity.

The environment plays a crucial role in interaction calculations. Existing studies have shown that incorporating the local environment of the binding pocket can significantly affect the strength of affinity (Wang 2024, Klebe 2025). For instance, Karlov *et al.* (2020) utilized distances and angles between atoms as environmental features. In contrast, Qu *et al.* (2024) and Zhang *et al.* (2024a) considered water as an important environmental factor, using water networks to characterize protein–ligand -water interactions. However, the environmental features currently employed are relatively simplistic and do not fully account for all potential influencing factors. There may be additional environmental features that could impact binding affinity, as illustrated in Fig. 1.

We propose a novel deep learning model, the Environment-aware Heterogeneous Graph-based Multi-modal protein–ligand Affinity Prediction Model (EM-PLA), designed to effectively capture the effects of protein and ligand atoms while separately computing non-covalent interactions. EM-PLA improves affinity prediction through three distinct modules: the protein module, which extracts features from the amino acid sequence; the sequence complex module, which utilizes a cross-attention mechanism (Vaswani, 2017) to compute interaction information, and the 3D complex module, which models the protein–ligand complex as a graph, distinguishing between covalent and non-covalent interactions to minimize interference while incorporating environmental features. EM-PLA was trained on the PDBBind dataset (Wang *et al.* 2005) and evaluated on multiple datasets, achieving superior performance compared to several state-of-the-art methods and demonstrating strong generalization. Ablation experiments confirmed the contributions of each individual module and feature, highlighting the effectiveness of EM-PLA in protein–ligand affinity prediction and its potential for neural network-based drug development. Key contributions of this work are as follows:

- We propose a multi-modal approach that integrates sequence and 3D complex information to enhance feature representation and computation rationality.
- A heterogeneous graph neural network model has been introduced to leverage the environmental features of both the protein and the ligand to assist in the calculation of non-covalent interactions.
- Through experiments, visual validation, and case studies, the excellent performance and generalizability of EM-PLA have been established.

## 2 Materials and methods

### 2.1 Datasets

The PDBbind 2016 dataset (Wang *et al.* 2005) is a widely used benchmark for protein–ligand binding affinity prediction. It includes experimentally determined binding affinities and structural data for protein–ligand complexes. The dataset is divided into three subsets: the general set (13 285 complexes), the refined set (4057 high-quality complexes), and the core set (290 selected complexes), with each set nested within the previous. In this study, we followed the data processing approach of Pafnucy (Stepniewska-Dziubinska *et al.* 2018), randomly selecting 1000 complexes from the refined set to form the validation set, with the remaining complexes included in the general set, resulting in 11 906 complexes for training.

To evaluate the generalization ability of our model, we used four test sets: CASF-2013 (195), Core-2016 (290), CSAR-HIQ (51), and CSAR-HIQ (36). CASF-2013 (195) was created by Li *et al.* (2014), and Core-2016 (290) is the core set of the 2016 PDBbind dataset (Liu *et al.* 2017). The CSAR-HIQ (51) and CSAR-HIQ (36) datasets (Dunbar *et al.* 2011) are part of the CSAR project, containing high-resolution 3D structures and experimentally determined binding affinities for 51 and 36 complexes, respectively. Since CSAR-HIQ lacks protein pocket files, we used those from the MBP (Yan *et al.* 2023). Furthermore, to prevent any data overlap between the training and testing sets, we utilized the validation method proposed by GEMS (Graber *et al.* 2024) to verify the four test sets, ensuring that there is no data leakage. For convenience, we refer to these datasets as Core-195, Core-290, CSAR-51, and CSAR-36.

### 2.2 Input representation

#### 2.2.1 Protein sequence input representation

In this paper, the protein sequence features are represented as $\mathrm{Pro}\in R^{1024\times 40}$, where 40 denotes the feature dimensions and 1024 indicates the maximum length, with zero padding applied for sequences shorter than this length. Among the 40-dimensional features, there are one-hot encodings of amino acids (21 dimensions), secondary structure element (SSE) information (8 dimensions) (Kabsch and Sander 1983), and physicochemical properties of residues (11 dimensions) (Jin *et al.* 2023).

#### 2.2.2 Sequence complex input representation

The sequence complex consists of two components: the pocket sequence and the ligand SMILES sequence. The pocket sequence features are represented as $\mathrm{Poc}\in R^{64\times 40}$, where 40 indicates the feature dimensions and 64 denotes the maximum length. The feature construction method is the same as described in Section 2.2.1 Protein sequence input representation. The ligand features are represented as $\mathrm{Lig}\in R^{150\times 18}$, where 18 denotes the feature dimensions and 150 indicates the maximum length, with zero padding applied as necessary. The 18-dimensional feature vector is generated using OpenBabel and includes atomic types (nine dimensions), atomic properties (four dimensions), and SMARTS matching patterns (five dimensions).

#### 2.2.3 3D complex input representation

The 3D complex consists of three parts: the protein pocket graph and ligand graph for calculating covalent interactions, and the complex graph for calculating non-covalent interactions.

The pocket graph is constructed with amino acids as the fundamental units and is represented as $G_{p}(V_{p},E_{p},X_{p})$, where $V_{p}\in R^{n\times 21}$ denotes the feature representation of the amino acids, utilizing one-hot vectors for the 21 types of amino acids as input. Here, *n* represents the number of amino acids. $E_{p}$ denotes the edge set, where edges are formed between amino acids that are within 5 Å of each other based on experimental findings. $X_{p}\in R^{n\times 3}$ represents the coordinate set of the amino acids.

The ligand graph is constructed with atoms as the fundamental units and is represented as $G_{l}(V_{l},E_{l},A_{l},X_{l})$, where $V_{l}\in R^{m\times 18}$ denotes the feature representation of the atoms, also generated using OpenBabel. Here, *m* represents the number of atoms. $E_{l}$ denotes the edge set. $A_{l}$ represents the edge features (12 dimensions), generated by OpenBabel, which include information such as bond lengths, bond angles, and bond types. $X_{l}\in R^{m\times 3}$ represents the coordinate set of the atoms.

Due to the non-covalent nature of interactions, the influence of surrounding atoms may have been overlooked. To mitigate this limitation, we introduced environmental nodes into the complex graph. The complex graph is a heterogeneous graph, with atoms as the fundamental units, comprising three types of nodes: proteins, ligands, and environments. The feature representations for the ligand and protein nodes are generated using OpenBabel, while the environmental nodes are composed of 11 biochemical indicators derived from proteins and ligands. These indicators include aromaticity, rotatable bonds, isoelectric points, hydrophilicity indexes, charge, topological polar surface area, octanol-water partition coefficients, as well as hydrogen bond donor and acceptor information for both proteins and ligands. Further details can be found in the Supplementary Data, available as supplementary data at *Bioinformatics* online.

Based on this, the overall representation of the complex is denoted as $G_{c}(V_{c},V_{e},E_{c},A_{c})$, where $V_{c}\in R^{N\times 18}$ represents the feature representations of the protein and ligand nodes, with *N* indicating the total number of protein and ligand nodes. $V_{e}\in R^{3\times 5}$ represents the feature representations of the environmental nodes. $E_{c}$ denotes the edge set, which includes three types of bidirectional edges (protein–ligand, environment–protein, and environment–ligand). $A_{l}$ represents the edge features (1-dimensional), with distance utilized as the edge feature. Considering that the environmental nodes are virtual but have a direct influence on other nodes, we set the distances between the environmental nodes and the protein and ligand nodes to zero.

### 2.3 The model architecture of EM-PLA

**Figure 2.**
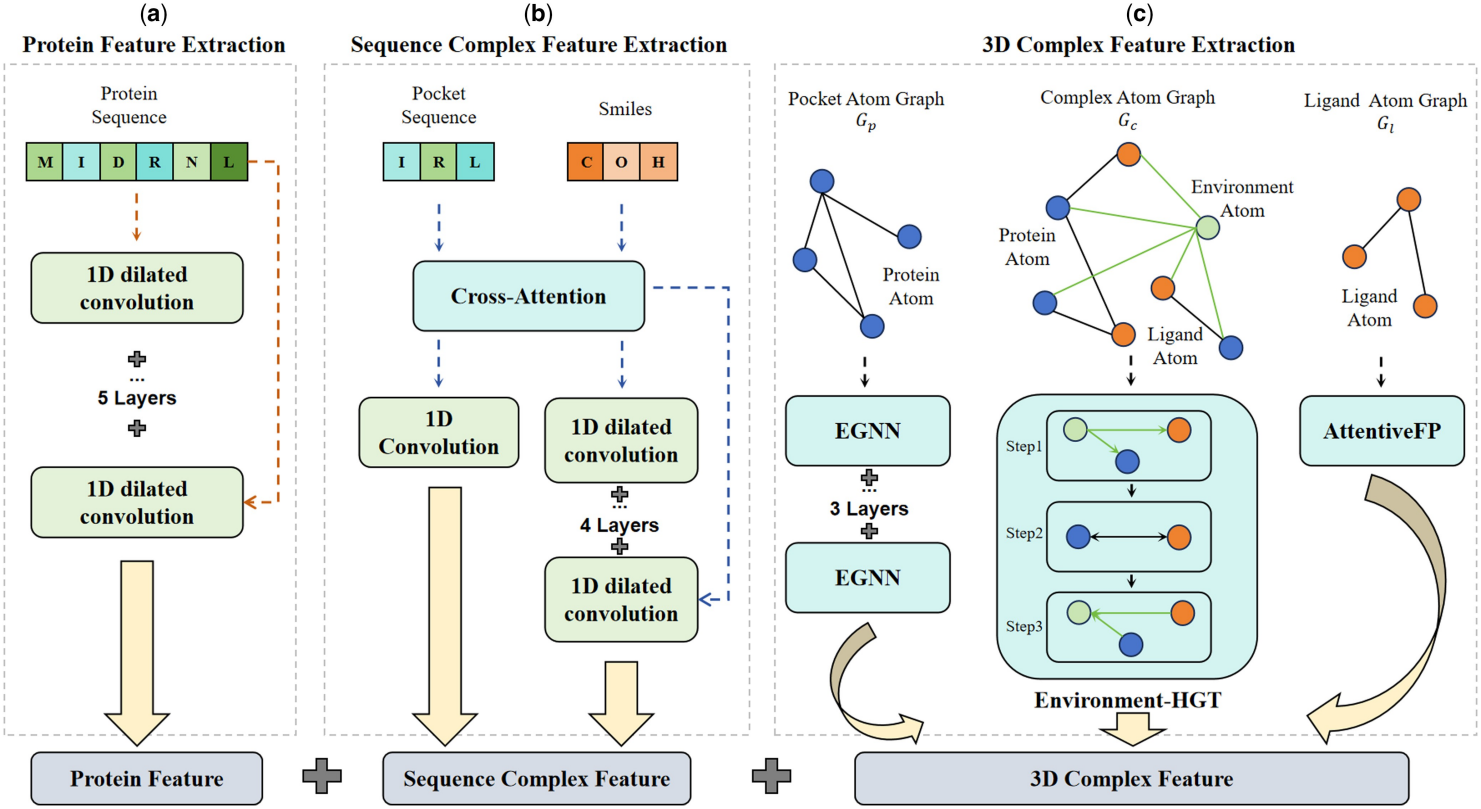
The overall architecture of the EM-PLA prediction framework is divided into three parts: (a) Protein Feature Extraction Module: extracts features from protein sequences. (b) Complex Sequence Feature Extraction Module: extracts interaction features between the binding pocket and ligand sequences. (c) 3D Complex Feature Extraction Module: extracts covalent interaction features using EGNN and AttentiveFP, and non-covalent interaction features with Environment-HGT, which considers environmental influences. In Environment-HGT, Step 1 calculates environmental effects on the complex, Step 2 assesses interactions within the complex, and Step 3 evaluates the impact of the complex on the environment.

The proposed model architecture is illustrated in Fig. 2. The model is mainly divided into three components: (i) Protein feature extraction module, (ii) Sequence complex feature extraction module, and (iii) 3D complex feature extraction module. These three modules will be introduced sequentially below.

#### 2.3.1 Protein feature extraction module

The complete sequence of a protein contains potential structural information and determines its three-dimensional structure. The input to this module is the protein sequence feature *Pro*. In this module, a one-dimensional dilated convolution model (Jin *et al.* 2023) is employed to extract protein sequence features. This method aggregates multi-scale contextual information by increasing the receptive field size with kernels of varying dilation rates. Based on this characteristic, the model is configured with five layers of dilated convolution, with dilation sizes of [1, 2, 4, 8, and 16]. These layers combine amino acid interaction features across different scales through concatenation. Specific parameters can be found in the Supplementary Data, available as supplementary data at *Bioinformatics* online.

#### 2.3.2 Sequence complex feature extraction module

Traditional methods primarily utilize sequence features and have achieved significant results, demonstrating the effectiveness of sequence-based representations. The input to this module includes the feature representations of the protein pocket sequence *Poc* and the ligand SMILES sequence *Lig*. Cross-attention is first applied to compute the interactions between the pocket and the ligand. The specific formulas are follows:


(1) $$\begin{array}{c} \mathrm{Attentio}n^{i}=\mathrm{Softmax}\left(\frac{Q_{i}K_{i}^{T}}{\sqrt{d_{k}}}\right)V_{i}\\ \mathrm{CrossAttention}=\mathrm{Concat}(\mathrm{Attentio}n^{1},\mathrm{Attentio}n^{2})W^{O} \end{array}$$

where, $Q_{i}$ represents the query vector for the *i*-th head, calculated as $QW_{i}^{Q}$. $K_{i}$ denotes the key vector for the *i*-th head, computed as $KW_{i}^{K}$. $V_{i}$ represents the value vector for the *i*-th head, calculated as $VW_{i}^{V}$. *Softmax* denotes the activation function, while *Concat* indicates the concatenation operation, and $W^{O}$ represents the output projection matrix. In this study, we first treat the pocket as *Q* and the ligand as *K* and *V* to compute the feature representation of the pocket. Subsequently, we treat the ligand as *Q* and the pocket as *K* and *V* to compute the feature representation of the ligand.

Subsequently, the enriched interaction feature representations are input into both a 1D CNN and a one-dimensional dilated convolution for further learning of their respective feature representations. In this context, the dilated convolution used for the ligands consists of four layers with dilation sizes of [1, 2, 4, and 8]. Similarly, the features of amino acid interactions at different scales are combined through concatenation.

#### 2.3.3 3D complex feature extraction module

In this study, we enhance our approach by separately calculating both covalent and non-covalent interactions while incorporating environmental features to provide a more comprehensive understanding of these interactions.

**Protein Covalent Interactions.** This module processes the protein pocket graph, where amino acid interactions are computed using the E(n) Equivariant Graph Neural Network (EGNN) (Satorras *et al.* 2021). EGNN is a graph neural network that can process 3D spatial information, and its equivariance enhances the ability to extract protein features. In this study, we employed a 3-layer EGNN architecture to extract features $P_{3d}$ from $G_{p}$:


(2) $$\begin{array}{c} V_{p}^{i+1},X_{p}^{i+1}=\mathrm{EGN}N^{i}\left(V_{p}^{i},X_{p}^{i}\right),\;i=0,1,2\\ P_{3d}=\mathrm{Concat}\left(V_{p}^{1},V_{p}^{2},V_{p}^{3}\right) \end{array}$$

where $V_{p}^{i}$ is the feature representation of the protein at the *i*-th layer, $X_{p}^{i}$ is the coordinate of the protein at the *i*-th layer, and *Concat* denotes the concatenation of feature vectors.

**Ligand Covalent Interactions.** The input to this module is the ligand graph $G_{l}$, and the interactions between atoms within the ligand are computed using the AttentiveFP (Xiong *et al.* 2020), this model is specifically designed for molecular feature extraction and can dynamically adjust the weights of different nodes and edges in the molecular graph. It effectively captures complex interactions within the molecule, including both local and non-local interactions, ultimately resulting in the ligand feature representation $L_{3d}$.

**Non-Covalent Interactions.** The spatial interactions between proteins and ligands are key to determining binding affinity, highlighting the importance of 3D structural considerations. This module takes the complex graph $G_{c}$ as input and employs an environment-based heterogeneous GNN (Zhang *et al.* 2019) for feature extraction. Unlike conventional heterogeneous graphs, which only model non-covalent interactions between atoms within a distance threshold, this design overlooks the broader atomic environment. Although covalent modules partially compensate for this, the approach remains limited and lacks interpretability. To address this issue, as described in Section 2.2.3 3D complex input representation, we introduced three environmental nodes. In addition, to more effectively utilize the environmental atoms, we designed a stepwise strategy for applying graph convolutions to the heterogeneous graph neural network. Specifically, the proposed environment-based heterogeneous graph convolutional network operates in three steps: (i) Calculate the impact of the environment on the complexes, and update the feature representations of the protein and ligand nodes. (ii) Calculate the non-covalent interactions and update the feature representations of the protein and ligand nodes. (iii) Calculate the impact of the protein and ligand on the environment, and update the feature representations of the environmental nodes. The process is detailed as follows:


(3) $$\begin{array}{cc} & \mathrm{Step}1:V_{c}^{i+1}=\mathrm{SAGECon}v_{e\_to\_c}^{i}(V_{c}^{i},V_{e}^{i},E_{e\_to\_c})\\ & \mathrm{Step}2:V_{c}^{i+1}=\mathrm{SAGECon}v_{c\_to\_c}^{i}(V_{c}^{i},E_{c\_to\_c})\\ & \mathrm{Step}3:V_{e}^{i+1}=\mathrm{SAGECon}v_{c\_to\_e}^{i}(V_{c}^{i},V_{e}^{i},E_{c\_to\_e}) \end{array}$$

where, $V_{c}^{i}$ represents the features of the protein and compound at the *i*-th layer, $V_{e}^{i}$ denotes the features of the environmental nodes at the *i*-th layer, and i=0,1,2. $E_{e\_to\_c}$ denotes the edge set from the environment to the proteins and ligands, $E_{c\_to\_c}$ represents the bidirectional edge set between proteins and ligands, and $E_{c\_to\_e}$ indicates the edge set from proteins and ligands to the environment. In this paper, SAGEConv (Hamilton *et al.* 2017) is used as the base convolution operation for the HGT. Finally, edge pooling and a multilayer perceptron (MLP) are employed to integrate the global embeddings of the heterogeneous graph and generate the final representation $C_{3d}$. More details can be found in the Supplementary Data, available as supplementary data at *Bioinformatics* online.

### 2.4 Loss functions and hyperparameters

The model was implemented in an environment with CUDA 11.3 and PyTorch 1.11.0, using mean squared error (MSE) as the loss function and the Adam optimizer for parameter optimization. Additionally, we utilized a Bayesian optimizer to explore the hyperparameter space and determine the optimal hyperparameters. Ultimately, the learning rate was set to 1e-4, with a learning rate decay factor of 0.1 and a patience of 10 epochs. The MSE loss function is defined as follows:


(4) $$L_{\mathrm{MSE}}=\frac{1}{N}\sum_{i=1}^{N}{\left(y_{i}-{\hat{y}}_{i}\right)}^{2}$$

where *N* is the total number of samples, $y_{i}$ represents the true value for the *i*-th sample, and ${\hat{y}}_{i}$ denotes the predicted value for the *i*-th sample. More details can be found in the Supplementary Data, available as supplementary data at *Bioinformatics* online.

### 2.5 Evaluation metrics

In this work, to thoroughly evaluate the performance of our proposed method and compare it with other baseline methods, we adopted five widely used evaluation metrics, including the Pearson correlation coefficient (R), mean Absolute Error (MAE), root mean square error (RMSE), standard deviation (SD), and concordance Index (CI).

## 3 Experimental results

In this section, we compare the EM-PLA method with state-of-the-art (SOTA) baseline methods across four benchmark datasets, demonstrating its superiority in predicting protein–ligand binding affinity. We further evaluated and analyzed the model in depth through ablation experiments, visualizations, and case studies.

### 3.1 Performance of EM-PLA and comparison with state-of-the-art methods

**Core-290 dataset.** The performance of EM-PLA on the Core-290 dataset, compared to baseline models, is shown in Table 1. Baseline model metrics come from their respective publications. DeepDTA, DeepDTAF, and CAPLA are sequence-based models, while others are structure-based. EM-PLA either matches or outperforms these models. DeepDTA and DeepDTAF struggle to capture protein–ligand interactions, leading to lower performance. CAPLA, with its cross-attention mechanism, performs better by modeling these interactions. Pafnucy uses 3D CNNs but lacks translation and rotation invariance, affecting its accuracy. OnionNet, while incorporating long-range interactions, fails to capture full protein information. IMCP-SF and MM-DRPNet use CNNs for 2D contact features, but reducing 3D data to 2D may result in information loss, reducing their performance. The MFE method (Xu *et al.* 2024) employs a heterogeneous graph neural network that integrates three-dimensional structural features with protein surface information. In light of its use of a heterogeneous graph neural network, we also conducted experiments incorporating environmental features, denoted as MFE-environment. However, the heavy reliance on protein sequences and surface data in MFE limits its ability to effectively capture interactions within protein–ligand complexes. In contrast, our method, EM-PLA, integrates the 3D structural information of complexes while also considering sequence-level complexes and holistic protein information. Additionally, it enhances the computation of non-covalent interactions by incorporating environmental information. This design achieves state-of-the-art performance while offering a more rational and interpretable approach.

**Table 1.** Performance on Core-290 dataset.

Method	R ↑	RMSE ↓	MAE ↓	SD ↓	CI ↑
DeepDTA	0.749	1.443	1.148	1.445	0.771
DeepDTAF	0.789	1.355	1.073	1.337	0.799
Pafnucy	0.775	1.418	1.129	1.375	0.789
OnionNet	0.816	1.278	–	1.45	–
IMCP-SF	0.791	1.452	1.155	1.349	0.79
CAPLA	0.843	1.200	0.966	1.170	0.820
MFE	0.851	1.151	0.882	1.138	–
MFE-Environment	0.854	1.154	0.897	1.134	0.833
MM-DRPNet	–	1.128	0.853	1.086	**0.884**
EM-PLA (Ours)	**0.875**	**1.055**	**0.827**	**1.055**	0.845

Bold numbers represent the best performance in each metric column.

**CASF-195 dataset.**  Table 2 compares the performance of EM-PLA with other baseline models on the CASF-195 dataset. The metrics for all other models are taken from their respective publications. Similar to its performance on the Core-290 dataset, models such as DeepDTAF, Pafnucy, and OnionNet do not perform well. The CAPLA method, which is based on sequence interactions, only considers single-modality features. MM-DRPNet incorporates multimodal features, including compound DRP features and ligand graphs. Our method further improves performance by considering both sequence complexes and 3D complexes, separately computing covalent and non-covalent interactions. EM-PLA achieves better performance and rationale, surpassing MM-DRPNet by 3.2% in the RMSE metric.

**Table 2.** Performance on CASF-195 dataset.

Method	R ↑	RMSE ↓	MAE ↓	SD ↓	CI ↑
DeepDTAF	0.608	2.103	1.737	1.787	0.717
Pafnucy	0.700	1.620	1.320	1.610	–
Onionnet	0.780	1.500	1.210	1.450	–
CAPLA	0.770	1.446	1.154	1.436	0.780
MM-DRPNet	–	1.212	**0.915**	1.198	**0.831**
EM-PLA (Ours)	**0.856**	**1.173**	0.962	**1.164**	0.829

Bold numbers represent the best performance in each metric column.

**CSAR-51 and CSAR-36.** To evaluate the generalization ability of EM-PLA, we further tested two external datasets (CSAR-51 and CSAR-36) that are independent of the previous test set. The results, as shown in Table 3, lead to the following observations:

**Table 3.** Performance on CSAR-36 and CSAR-51 datasets.

Method	CSAR-36					CSAR-51				
	R ↑	RMSE ↓	MAE ↓	SD ↓	CI ↑	R ↑	RMSE ↓	MAE ↓	SD ↓	CI ↑
DeepDTAF	0.543	2.765	2.318	1.679	0.670	0.606	2.272	1.862	1.860	0.710
Pafnucy	0.566	1.658	1.291	1.649	0.566	0.622	1.944	1.667	1.832	0.698
IGN	0.528	1.795	1.431	1.699	0.676	0.417	2.263	1.714	2.125	0.657
IMCP-SF	0.631	1.560	1.205	1.573	0.748	**0.769**	**1.629**	**1.278**	**1.491**	**0.780**
CAPLA	0.704	1.454	1.160	1.420	0.760	0.686	1.848	1.550	1.701	0.727
EM-PLA (Ours)	**0.780**	**1.246**	**0.995**	**1.253**	**0.794**	0.709	1.651	1.356	1.653	0.735

Bold numbers represent the best performance in each metric column.

On the CSAR-36 dataset, both Pafnucy (CNN-based) and IGN (Li *et al.* 2021) (GNN-based) performed poorly, while 2D contact-based IMCP-SF and sequence-based CAPLA achieved better results. However, our model outperformed them by 20.1% and 14.3% in RMSE, respectively. On the CSAR-51 dataset, 3D-based methods were suboptimal, while CAPLA performed better. Our model outperformed CAPLA by 10.7% in RMSE, though slightly lagged behind IMCP-SF.

This comparison highlights that methods relying on low-dimensional features exhibit superior generalization compared to those based on 3D complexes. Possible reasons for this include: (i) Low-dimensional feature-based methods often incorporate more comprehensive protein features due to their lower computational cost, thereby enhancing generalization ability. (ii) Interaction features of 3D complexes are not effectively captured during feature extraction, limiting the performance of such models. (iii) Both 3D-CNNs and heterogeneous graph neural networks fail to account for translational and rotational invariance. While our method leverages EGNN to extract covalent interactions in proteins, the invariance of non-covalent interactions remains unaddressed. As a result, similar complexes may exhibit significant differences, which reduces generalization performance.

In summary, our method shows strong applicability and generalization in protein–ligand binding affinity prediction, outperforming state-of-the-art baseline methods across four benchmark datasets.

### 3.2 Ablation study

To demonstrate the effectiveness and necessity of complete protein information, sequence complexes, 3D complexes, and environmental information, we conducted the following ablation experiments on the Core-290 and CSAR-36 datasets: (i) W/O environmental information: the environmental nodes within the non-covalent interactions are removed. (ii) W/O covalent interactions: the covalent interaction components, including protein covalent interactions(EGNN) and ligand covalent interactions(AttentiveFP), are removed. (iii) W/O non-covalent interactions: the non-covalent interaction components (HGT) are removed. (iv) W/O sequence complexes: the sequence complex feature extraction module is removed. (v) W/O protein information: the protein feature extraction module is removed.

Table 4 displays five metrics under different settings on the Core-290 and CSAR-36 datasets. The CSAR-36 dataset allows for the analysis of the impact of different modules on the generalization performance of the model.

**Table 4.** Ablation study on core-290 and CSAR-36 datasets.

Ablation setting	Core-290					CSAR-36				
	R ↑	RMSE ↓	MAE ↓	SD ↓	CI ↑	R ↑	RMSE ↓	MAE ↓	SD ↓	CI ↑
W/O environmental information	0.829	1.245	0.952	1.219	0.815	0.734	1.405	1.154	1.358	0.781
W/O covalent interactions	0.820	1.250	0.959	1.246	0.816	0.651	1.503	1.194	1.519	0.754
W/O non-covalent interactions	0.822	1.254	0.959	1.240	0.812	0.638	1.618	1.236	1.540	0.738
W/O sequence complexes	0.806	1.296	1.014	1.288	0.806	0.671	1.887	1.420	1.483	0.765
W/O protein information	0.822	1.257	0.985	1.239	0.809	0.617	1.559	1.268	1.574	0.735
Full	**0.875**	**1.055**	**0.827**	**1.055**	**0.845**	**0.780**	**1.246**	**0.995**	**1.253**	**0.794**

Bold numbers represent the best performance in each metric column.

**Figure 3.**
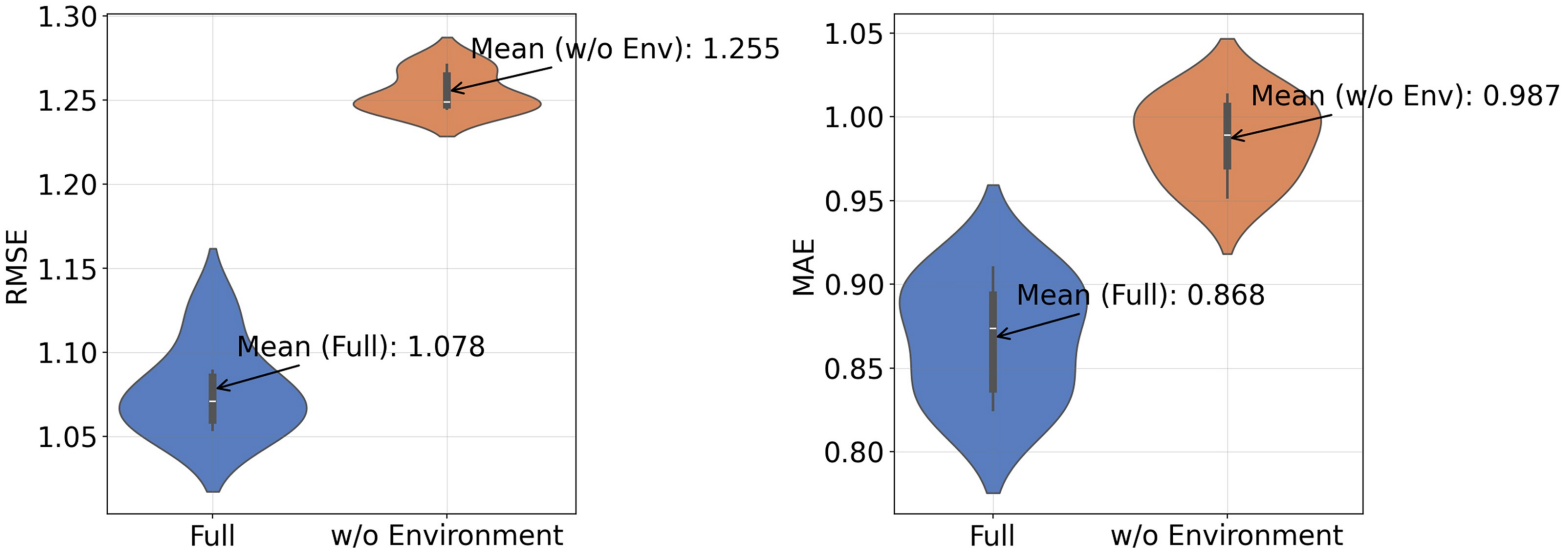
Violin plots of the RMSE and MAE metrics for the W/O environmental information experimental setting and the full model on the Core-290 dataset.

From the results of the ablation study, we can analyze that: (i) The model primarily focuses on interactions rather than favoring proteins or ligands (without considering generalization). In TransformerCPI (Chen *et al.* 2020), it was observed that some models exhibited a tendency to rely heavily on a single module feature, while in our model, on the Core-290 dataset, the RMSE metric decreased by a maximum of 18.6% and a minimum of 15.3% when different modules were removed, indicating that the model’s dependence on different modules is relatively balanced. (ii) Interactions can enhance generalization ability. The impact of different experimental settings on the Core-290 dataset is relatively small, but it is more pronounced on the independent CSAR-36 dataset. We hypothesize that this is due to the varying influence of different modules on the model’s generalization ability. Furthermore, it can be observed that on the CSAR-36 dataset, the two most impactful modules are W/O sequence complexes and W/O non-covalent interactions. The absence of sequence interactions or 3D interactions leads to a significant decline in the model’s generalization ability, suggesting that considering interactions in the model can better enhance generalization performance. (iii) Environmental information is crucial. Figure 3 shows the distribution of RMSE and MAE metrics for the full model and the W/O environmental information setting on Core-290 dataset. As seen in the figure, the performance decline due to the absence of environmental information is evident across multiple experiments, with both RMSE and MAE metrics lower than those of the full model. This suggests that incorporating environmental information enhances interaction calculations by accounting for indirect effects from other atoms.

### 3.3 Visualization

**Figure 4.**
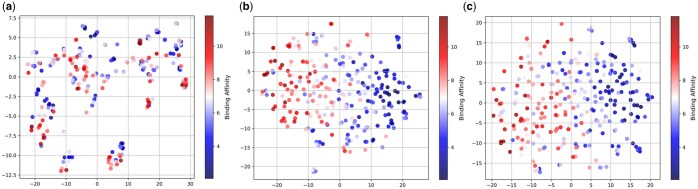
Visualization of feature representation distribution using t-SNE under three conditions: (a) initial input embedding representation, (b) embeddings generated by the model without environmental nodes, and (c) embeddings generated by the model with environmental nodes.

In this section, we visualize the embeddings of the model both with and without environmental nodes. Specifically, we performed the visualization on all complexes in the CASF-195 test set to analyze the impact of environmental nodes. Using the t-SNE method, we reduced the dimensionality of the embeddings generated by the model for predicting binding affinity and created visual representations, as illustrated in Fig. 4. Figure 4a represents the initial input embeddings, Fig. 4b depicts the embeddings generated without environmental nodes, and Fig. 4c shows the embeddings generated with environmental nodes.

It can be observed that in Fig. 4a, the complexes with different binding affinities overlap after t-SNE dimensionality reduction, making them indistinguishable. At this stage, the embedding representation is insufficient to effectively support the ligand-protein binding affinity prediction task. In Fig. 4b, the embeddings obtained using a general heterogeneous graph for feature extraction, without environmental features, can distinguish complexes with different binding affinities fairly well. However, compared to Fig. 4c, it still exhibits limitations. In Fig. 4c, the boundary distinctions are much clearer, with a reduced degree of overlap between the data points representing protein-ligand pairs of different binding affinities. Additionally, in Fig. 4b, there are still significant instances where low-affinity complexes are misclassified as high-affinity complexes. In contrast, this issue is alleviated in Fig. 4c, indicating that the model is better able to differentiate between complexes of varying binding affinities. These results demonstrate that the inclusion of environmental nodes enables the model to learn more distinctive embeddings for the complexes, thereby enhancing the performance of binding affinity predictions.

### 3.4 Case study

In this section, we conduct a detailed case study using the EM-PLA model. The specific method involves applying the t-SNE technique to reduce the dimensionality of the node embeddings in the heterogeneous graph, allowing us to observe the spatial distribution of environmental nodes, protein nodes, and ligand nodes. This dimensionality reduction enables us to identify the relationships between nodes, a shorter distance indicates a stronger correlation between the embeddings. Additionally, we used PyMol to visualize the 3D structure of the complex, identifying the key amino acids and atoms.

**Figure 5.**
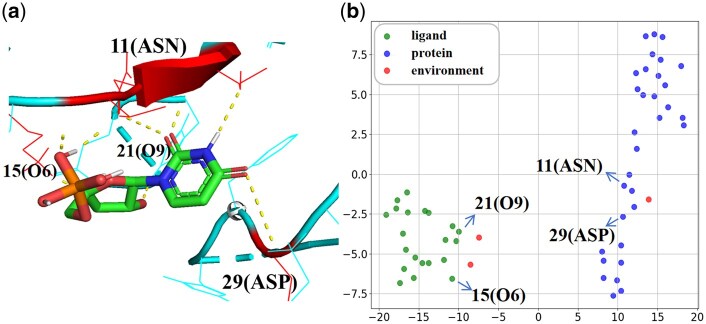
Case study of the 3dxg complex. (a) 3D structure of the complex generated by PyMol. (b) Scatter plot of all nodes in the environmental heterogeneous graph, visualized using the t-SNE method.

Figure 5 presents a case study of the complex 3dxg. Figure 5a displays the complex structure generated by PyMOL, which includes two protein binding regions: amino acid 11 (ASN) and amino acid 29 (ASP). Figure 5b presents a scatter plot of the protein–ligand -environment node representations, created by the t-SNE method. In this plot, the three environmental nodes are distributed around the ligand nodes and protein nodes. The protein nodes that are close to the environmental nodes include the key amino acids (11(ASN) and 29(ASP)), while the ligand nodes close to the environmental nodes contain two of the key atoms (15(O6) and 21(O9)).

These results indicate that the environmental nodes in the heterogeneous graph can focus on the key residues of the protein or the critical atoms of the ligand. This learning significantly impacts the computation of protein–ligand interactions, thereby enhancing the accuracy of affinity predictions. Additional case studies can be found in the Supplementary Data, available as supplementary data at *Bioinformatics* online.

## 4 Conclusion and future work

In this work, we developed a novel deep learning method, EM-PLA, based on multimodal complexes for protein–ligand binding affinity prediction. Our experiments on various benchmark datasets demonstrated the effectiveness and generalization ability of the method on independent test sets. The ablation study validated that our method does not overly depend on any single type of feature, and visualizations along with case studies demonstrated that incorporating environmental information can enhance the model’s perceptual capabilities.

However, there are areas for improvement. Heterogeneous graph neural networks currently lack equivariance, therefore exploring the combination of equivariant properties with environmental information could enhance the model’s perceptual capabilities for a broader range of structures. Furthermore, the potential applications of EM-PLA in drug discovery require further exploration.

## Supplementary Material

btaf298_Supplementary_Data
