# Supplementary material for: EM-PLA: environment-aware heterogeneous graph-based multimodal protein–ligand binding affinity prediction
Source: Bioinformatics. 2025 May 12;41(7):btaf298. doi: 10.1093/bioinformatics/btaf298 (PMC12255879; doi:10.1093/bioinformatics/btaf298)
Supplement: btaf298_Supplementary_Data [file btaf298_supplementary_data.docx]

**1 The validation method with non-overlapping datasets**

Specifically, we employed the training-testing pair screening method proposed by GEMS to revalidate our four test sets. The GEMS approach can be summarized as follows: training-testing pairs will be removed if they meet the following three conditions:

1. The proteins have a TM-score higher than 0.8
2. The sum of the Tanimoto score (T) and the inverted RMSD is higher than 0.8 *(T + (1 − RMSD))
3. The affinity labels are similar (±1 in pK units)

In the GEMS method, Tanimoto similarity is calculated using RDKit; however, a subset of compounds could not be processed by RDKit. For this subset, we utilized Pybel to compute the Tanimoto similarity.

Through our validation, we confirmed that all our training-testing pairs meet the specified criteria, ensuring that there is no overlap in the data.

Table 1. Assessment of the overlap between the training and testing sets using the GEMS method.

| Training set | Testing set | Training-testing pairs that do not meet GEMS standards |
| --- | --- | --- |
| PDBBind-2016 (11,906) | CASF-2013 (195) | None |
| PDBBind-2016 (11,906) | Core-2016(290) | None |
| PDBBind-2016 (11,906) | CSAR-HIQ (51) | None |
| PDBBind-2016 (11,906) | CSAR-HIQ (36) | None |

**2 Method for Constructing Environmental Features**

We selected a total of 11 biochemical features for the proteins and compounds, as detailed in the table below, where 0 represents zero padding.

Table 2. Specific representations of the three environmental nodes.

| 1 | Aro，NRB，0，0，0 |
| --- | --- |
| 2 | pI，GRAVY，Q7.4，HBD_seq，HBA_seq |
| 3 | TPSA，LogP，HBD_mol，HBA_mol,0 |

The first group includes the aromaticity of the protein (Aro) and the number of rotatable bonds in the compound (NRB). These two properties represent the structural dynamics of the protein and compound in three-dimensional space. For instance, the structure of aromatic rings is generally stable, while chemical reactions can alter the number of rotatable bonds, subsequently changing the overall structure.

The second group comprises five properties: the isoelectric point of the protein (pI), the GRAVY index of the protein (GRAVY), the charge of the protein at pH 7.4 (Q7.4), the number of hydrogen bond donors in the protein sequence (HBD seq), and the number of hydrogen bond acceptors in the protein sequence (HBA seq). These properties reflect the physicochemical characteristics of the protein.

The third group includes four properties: the polar surface area of the compound (TPSA), the LogP value of the compound (LogP), the number of hydrogen bond donors in the compound (HBD mol), and the number of hydrogen bond acceptors in the compound (HBA mol). These properties represent the physicochemical characteristics of the compound.

Table 3. Rough estimation of potential donor and acceptor amino acids.

| Amino Acid Name | Three-Letter Abbreviation | One-Letter Abbreviation | Side Chain Functional Group |
| --- | --- | --- | --- |
| Lysine | Lys | K | ε-Amino group (NH₃⁺) |
| Arginine | Arg | R | Guanyl group (NH₂⁺) |
| Histidine | His | H | Imidazole NH group |
| Serine | Ser | S | Hydroxyl group (OH) |
| Threonine | Thr | T | Hydroxyl group (OH) |
| Tyrosine | Tyr | Y | Hydroxyl group (OH) |
| Glutamic Acid | Glu | E | Carboxyl group (COO⁻) |
| Aspartic Acid | Asp | D | Carboxyl group (COO⁻) |
| Asparagine | Asn | N | Amide carbonyl (C=O) |
| Glutamine | Gln | Q | Amide carbonyl (C=O) |

**3 Model Parameter Configuration**

Table 4. Model Parameter Configuration

| Hyperparameter | Value | |
| --- | --- | --- |
| Protein Sequence Feature Extraction Module - 1D Dilated Convolution (Repeated Four Times, Output Dimensions: [32, 64, 64, 128]) | Layer 1 | Dilation rates =1，kernel size=3，padding=1 |
|  | Layer 2 | Dilation rates =2，kernel size=3，padding=2 |
|  | Layer 3 | Dilation rates =4，kernel size=3，padding=4 |
|  | Layer 4 | Dilation rates =8，kernel size=3，padding=8 |
|  | Layer 5 | Dilation rates =16，kernel size=3，padding=16 |
| Pocket Sequence Feature Extraction in the Sequence Complex Feature Extraction Module - 1D Convolution (Repeated Three Times, Output Dimensions: [32, 64, 128]) | Layer 1，Layer 2，Layer 3 | padding =1，kernel size=3 |
| Compound SMILES String Feature Extraction in the Sequence Complex Feature Extraction Module - 1D Dilated Convolution (Repeated Three Times, Output Dimensions: [32, 64, 128]) | Layer 1 | Dilation rates =1，kernel size=3，padding=1 |
|  | Layer 2 | Dilation rates =2，kernel size=3，padding=2 |
|  | Layer 3 | Dilation rates =4，kernel size=3，padding=4 |
|  | Layer 4 | Dilation rates =8，kernel size=3，padding=8 |
| Pocket 3D Feature Extraction in the 3D Complex Feature Extraction Module - EGNN | Layer 1 | n_layers=12，edge_features=0，  out_node_nf=128 |
|  | Layer 2 | n_layers=12，edge_features=0，  out_node_nf=64 |
|  | Layer 3 | n_layers=12，edge_features=0，  out_node_nf=32 |
| Compound 3D Feature Extraction in the 3D Complex Feature Extraction Module - AttentiveFP | hidden_channels=64,  out_channels=16,  edge_dim=12,  num_timesteps=3,  num_layers=3, | |
| Complex 3D Feature Extraction in the 3D Complex Feature Extraction Module - HGT | edge_mlps的MLP | 132，512，64，16 |
|  | edge_lins的Linear | in_channels=1, out_channels=8 |
|  | Convs (Environment Nodes to Other Nodes) | hidden_channels=64  num_layers = 3 |
|  | Convs (Node to Node, Excluding Ring Nodes) |  |
|  | Convs (Other Nodes to Environment Nodes) |  |
| Dropout in MLP for Affinity Prediction | 0.3 | |
| Dropout in AttentiveFP | 0.3 | |

**4 Heterogeneous Graph Convolution Process**

First, we perform convolution operations on the two types of edges: [ ('env', 'to', 'protein'), ('env', 'to', 'ligand') ]. The convolution calculations for each type of edge are as follows:

$$H_{i}^{l+1}=\mathrm{AGGREGATE}_{j\in N(i)}(H_{j}^{l})W$$

where, $N(i)$ represents the neighboring nodes, $H_{j}^{l}$ denotes the feature representation of the j-th node at layer $l$, and $\mathrm{AGGREGATE}$ indicates the aggregation operation. In this study, we employ mean aggregation.

Next, we perform convolution calculations on the two types of edges: [ ('ligand', 'to', 'protein'), ('protein', 'rev_to', 'ligand') ]. The convolution calculations for each type of edge are identical to those described previously:

$$H_{i}^{l+1}=\mathrm{AGGREGATE}_{j\in N(i)}(H_{j}^{l})W$$

Next, we perform convolution calculations on the edges [ ('protein', 'rev_to', 'env'), ('ligand', 'rev_to', 'env') ], with the following formula:

$$H_{i}^{l+1}=\mathrm{AGGREGATE}_{j\in N(i)}(H_{j}^{l})W$$

$$H_{i}^{l+1}=Combine(H_{i}^{l+1})$$

The operation $\mathrm{Combine}$ denotes the combination operation, and we choose the sum method for this purpose. In contrast to the previous steps, this step introduces the $\mathrm{Combine}$ operation, which leverages the characteristics of heterogeneous graph convolution. Specifically, if multiple edges share the same target node, the various feature representations for that target node are combined to generate the final feature representation.

Based on the above three steps, we have implemented the convolution computation for the environment-aware heterogeneous graph, resulting in updates to the environment nodes, protein nodes, and compound nodes.

**5 Validation of Environmental Features**


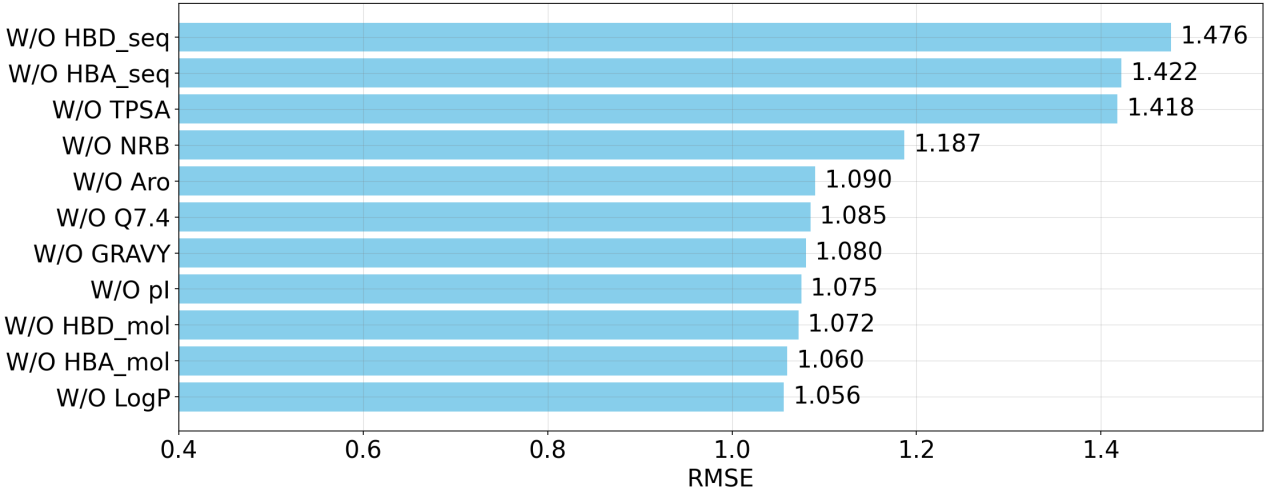


Figure. 1. A bar chart illustrating the ablation experiments of 11 environmental features on the Core-290 dataset.

Figure 1 further validates the importance of environmental features by displaying the RMSE metrics of the model on the Core-290 dataset after the removal of 11 environmental features (with only one type removed per experiment). Almost all environmental features significantly impact the model's performance.

**6 Validation of Key Modules of the Model**


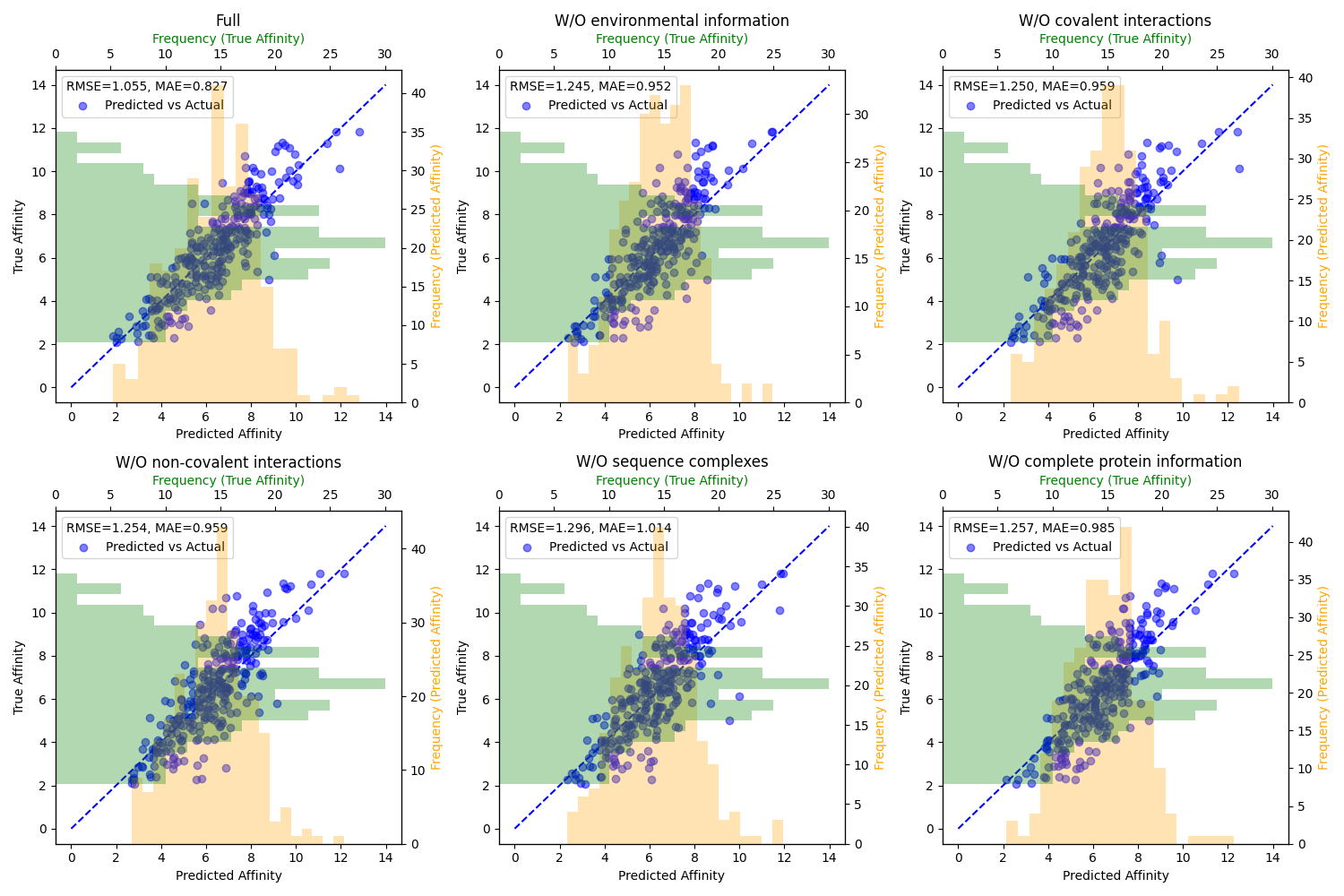


Figure. 2. The ablation study results on the Core-290 dataset include: (a) Full. (b) W/O environmental information. (c) W/O covalent interactions. (d) W/O non-covalent interactions. (e) W/O sequence complexes. (f) W/O protein information.

Figure 2 illustrates the affinity prediction distributions under different ablation experiment settings. It is evident that the complete model's predicted affinity distribution is much closer to the true values, while other methods show varying degrees of deviation, with the W/O non-covalent interactions setting exhibiting a relatively greater divergence. This result also indicates that our approach does not solely rely on features from any single modality.

**7 Additional Case Study**


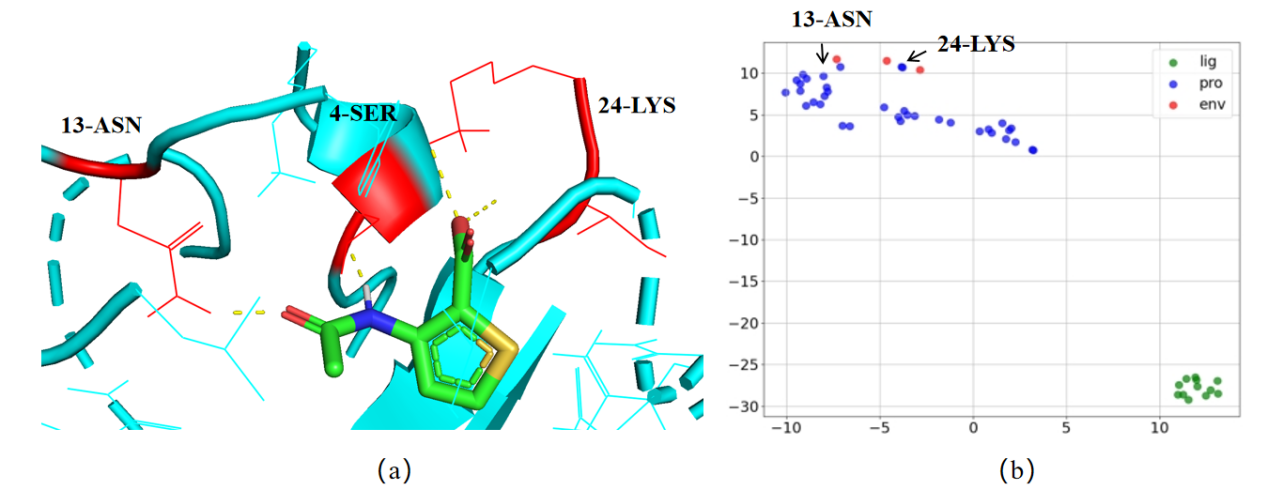


Figure. 3. Case study of the 3gv9 complex. (a) 3D structure of the complex generated by PyMol. (b) Scatter plot of all nodes in the environmental heterogeneous graph, visualized using the t-SNE method.
